# Plant Defense Genes against Biotic Stresses

**DOI:** 10.3390/ijms19082446

**Published:** 2018-08-19

**Authors:** Isabel Diaz

**Affiliations:** 1 Centro de Biotecnología y Genomica de Plantas (CBGP, UPM-INIA), Universidad Politéecnica de Madrid (UPM)—Instituto Nacional de Investigacion y Tecnología Agraria y Alimentaria (INIA), Campus de Montegancedo, Pozuelo de Alarcon, 28223 Madrid, Spain; i.diaz@upm.es; 2 Departamento de Biotecnologia y Biologia Vegetal, Escuela Tecnica Superior de Ingenieria Agronomica, Alimentaria y de Biosistemas, UPM, 28040 Madrid, Spain

The study of plant–biotic stress interactions is an exciting and fast-moving research field built around a dialog concerning plants with other alive organisms. In the last decades, it has offered extensive literature on the plant-pathogen relationship and, more recently, has produced new information on the battle between plants and arthropod-pests. The fact that higher plants are sessile organisms has favoured the acquisition of sophisticated resources to prevent or hamper biotic stresses either mediated by pathogens or pests. These relationships are an intricate interaction encompassing complex networks of molecules, signalling pathways, and strategies to overcome defences developed by each other. The induction of plant defence genes is initiated when specific receptors recognize either the presence of a pathogen (fungi, bacteria, and viruses), or a pest (phytophagous insects, acari, or nematodes), or the damage incurred by them, or even the existence of volatiles, emitted as plant-plant cues. Progress has been made in the molecular analyses of the plant defence system against biotic stresses, revealing a plethora of key elements, regulators, and pathways associated with these complex plant networks [1,2].

This special issue, along with 17 research papers and 6 reviews, gives a wide and actual perspective of this subject (Table 1).

Two reviews clarify important basic concepts such as tolerance and resistance [3], or elicitor and effector meaning [4]. In particular, Pagan and Garcia-Arenal [3] summarize the current theories and experimental evidence on the evolutionary causes and consequences of plant tolerance to pathogens and the existing knowledge on the genetic determinants and mechanisms of tolerance. The work indicates that tolerance may be as important as resistance in determining the dynamics of plant–pathogen interactions. Santamaria et al. [4] deal with the early plant recognition of pest molecules (elicitors and effectors) to assess fast plant responses by triggering a wide range of specific genes and compounds with defence properties. They present an actual landscape of the diverse strategies employed by plants within the first hours after pest perception to block the capability of phytophagous insects to develop mechanisms of resistance. 

Two other reviews are focused on specific players involved in plants’ resistance to pathogens: (i) host proteases located at different subcellular compartments that act as integral enzymes of the plant immune system and regulate hypersensitive response, pathogen recognition, priming, and peptide hormone release [5] and (ii) NOD-like receptors, one of the largest gene families in plants subjected to rapid evolution, that can induce an autoimmunity state that strongly affects plant growth and yield [6].

Regarding plant-virus interactions, Rong et al. [7] provide interesting data on molecular and ultrastructural mechanisms underlying Yellow Dwarf symptom formation in virus-infected wheat with barley Yellow Dwarf. A nice study of the chloroplast ultrastructure via transmission electron microscopy, together with comparative transcriptome through microarray analyses in a virus-susceptible wheat line, demonstrates that the virus symptoms are mainly attributed to reduced chlorophyll content caused by a precise transcriptional regulation of genes involved in chloroplast biosynthesis, ABA- and ethylene signalling-, and ROS-related genes. In addition, it is well-known that RNA silencing is a conserved mechanism that participates in RNAi-mediated host immunity associated with viruses. Qin et al. [8] decipher the main components (Argonaute and Dicer-like proteins, and RNA-dependent RNA polymerase) of the RNA silencing in virus infected-pepper. This study is focused on some of these up-regulated component genes induced by several virus infections and provides useful information for the elucidation of RNA silencing pathways.

Cabrera et al. [9] illustrate how gene expression in the nematode-feeding sites relates to morphological features by using a method based on green auto-fluorescence produced by glutaraldehyde and the tissue-clearing properties of benzyl-alcohol/benzyl-benzoate that preserves the structure of the nematode-feeding sites. Combining optical sections with confocal microscopy allows for the visualization and imaging of gall in thin (*Arabidopsis*) and thick (cucumber) nematode infested-roots, and for one to quantify and compare feeding cells size in a wild-type and an overexpression line. Additionally, within the plant-biotic stress mediated by nematodes, Zhao et al. [10] show that soluble sugar content was increased in tomato leaves during early infection by root-knot nematodes as a result of the activation of photosynthesis or the hydrolysis of starch, whereas an increase in sugar in the roots is a consequence of sugar translocation from source to sink tissue via transporters. Thus, authors determine the function of sugar transporter genes in infected-tomato. Post-infection responses in some sucrose transporter-mutant and overexpressing lines add information about the importance of sugar during early infection.

Two papers, a review and a research article, deal with the plant stress induced by phytophagous spider mites. Paulo et al. [11] analyse the ability of three mite species to suppress plant defences in Solanaceae, Convolvulaceae, and Fabaceae plants. The study suggests that the suppression of plant defences is not associated with adaptation to host plants. Besides, Agut et al. [12] review the most recent findings on plant defence mechanisms against the two-spotted spider mite *Tetranychus urticae*, an extremely polyphagous species found worldwide. Authors discuss different plant–mite interactions that are relative to the level of adaptation of the mite and the plant’s defence response, and conclude that induced resistance against mites is an interesting approach to combine with other existing IPM tools.

In relation to other methodological aspects, some reports are focused on the mapping of genes and QTL associated with pathogen resistance derived from intra-specific crosses, marker assisted molecular profiling, and RNA-seq assays [13,14,15,16]. The aim is to identify chromosome regions, transcriptomic locations of interest, and specific genes of resistance against phytopathogenic fungus and bacteria, developed in common bean, eggplant, tomato, and wheat, although most results are still preliminary, provide insights into the genetic basis of resistance, and suggest molecular strategies for breeding cultivars resistant pathogens. In addition, a genome-wide investigation on antimicrobial peptide (AMP) genes in maize genome developed by Noonan et al. [17] reveals that the polymorphisms of AMP genomic sequences in maize are valuable as new natural plant resistance sources to confer fungus and insect resistance for maize breeding applications in maize.

The use of plant transgenesis through the integration of a desirable gene in the genome of a crop species may confer resistance to a specific pathogens and pests. Based on this premise, seven articles propose this alternative approach to generate enhanced plants to control pathogens and pests. Li et al. [18] report the transient overexpression of *HvSERK2*, a barley somatic embryogenesis receptor-like kinase, in epidermal cells improved barley resistance to powdery mildew caused by *Blumeria graminis*, possibly though the H_2_O_2_ signalling pathway. Likewise, the overexpression of *VpRPW8s*, a typical R gene-mediated defence from a wild grapevine, enhances resistance to *Phytophtora capsici* in tobacco. Overexpressed *VpRPW8s* genes, encoding the nucleotide-binding site (NB) domains and/or the leucine-rich repeats (LRR), enhance resistance to *P. capsici* and confer varying degrees of resistance to *P. capsici* in *Nicotiana benthamiana* [19]. The ectopic expression of an ethylene response transcription factor (*VaERF20*) from a Chinese wild *Vitis* genoptype in the model plant *Arabidopsis thaliana* displays enhanced resistance to *Botrytis cinerea* and to the bacterium *Pseudomonas syringae* pv. tomato DC3000 by activating salycilic acid and jasmonic acid/ethylene signaling [20]. A chitinase gene from *Eucommia ulmoides (EuCHIT2)*, expressed in tobacco, confers resistance to *Erysiphe cichoracearum* infection [21]. However, the resistance mechanism of this gene is still unknown and requires further research. Similarly, three more articles pay attention to this biotechnological tool to control phytophagous aphids and mites. Losvik et al. [22] study the overexpression and down-regulation of the lipoxygenase gene *LOX2.2* in barley on the performance of two aphid species, the generalist *Myzus persicae* and the specialist *Rhopalosiphum padi*. They demonstrate that LOX2.2 plays a role in the activation of JA-mediated responses. The same research group publishes the use of the barley serine protease inhibitor *CI2c*, as transgene, and the inhibition of *M. persicae* fecundity when these aphids are fed on *CI2c*-Arabidopsis transgenic plants compared to control plants. The transgene probably inhibits aphid metabolism or unknown aspects of its reproduction [23]. Likewise, since the inhibitory role of barley HvCPI-6 cystatin (*Icy6* gene), a cysteine protease inhibitor, against the phytophagous mite *T. urticae* had been previously demonstrated, Santamaria et al. [24] generated *HvIcy6*-barley overexpressing plants and found significant lower damage area and lower presence of the mite than control infested plants. Transcriptomic analysis of these transgenic plants reveals a certain reprogramming of cellular metabolism and a lower expression of several genes related to photosynthetic activity. 

Finally, an excellent review reported by Singh et al. [25] discusses all aspects of the molecular biology of disease resistance in rice. It includes the general approaches used for developing resistant plants, the genetic basis of host resistance, signal transduction pathways, defence mechanism, and regulation of defence mechanism by regulatory elements; their perspectives in rice resistance will be discussed in response to fungal, bacterial, and viral pathogens.

Although substantial progress has been made in identifying plant defence genes against biotic stresses, as is shown here, further research is required to obtain a full understanding of how plants integrate defence responses, which is essential for exploiting natural defence mechanisms in agriculture.

## Figures and Tables

**Table 1:** Summary of the publications in this special issue.

Publication Title	Ref.
Tolerance to plant pathogens: theory and experimental evidence	[3]
Plant perception and short-term responses to phytophagous insects and mites	[4]
Ten prominent host proteases in plant-pathogen interactions	[5]
Regulation and evolution of NLR genes: a close interconnection for plant immunity	[6]
Molecular and ultrastructural mechanisms underlying yellow dwarf symptom formation in wheat after infection of Barley Yellow Dwarf Virus	[7]
Genome-wide analysis of DCL, AGO, and RDR gene families in pepper (*Capsicum annuum* L.)	[8]
A phenotyping method of giant cells from root-knot nematode feeding by confocal microscopy highlights a role for CHITINASE-like 1 in *Arabidopsis*	[9]
The role of sugar transporter genes during early infection by root-knot nematodes	[10]
Suppression of plant defences by herbivorous mites is not associated with adaptation to host plants	[11]
Can plant defence mechanisms provide new approaches for the sustainable control of the two-spotted spider mite *Tetranychus urticae*?	[12]
Mapping quantitative trait loci (QTL) for resistance to Late Blight in tomato	[13]
Genotyping by sequencing highlights a polygenic resistance to *Ralstonia pseudosolanacearum* in eggplant (*Solanum melongena* L.)	[14]
Marker-assisted molecular profiling, deletion mutant analysis, and RNA-Seq reveal a disease resistance cluster associated with *Uromyces appendiculatus* infection in common bean *Phaseolus vulgaris* L.	[15]
Transcriptome analysis identifies a 140 kb region of chromosome 3B containing genes specific to *Fusarium* Head Blight resistance in wheat	[16]
Investigation of antimicrobial peptide genes associated with fungus and insect resistance in maize	[17]
Transient overexpression of *HvSERK2* improves barley resistance to Powdery Mildew	[18]
Molecular characterization and overexpression of VpRPW8 from *Vitis pseudoreticulata* enhances resistance to *Phytophtora capsici* in *Nicotiana benthamiana*	[19]
Expression of *Vitis amurensis VaERF20* in *Arabidopsis thaliana* improves resistance to *Botrytis cinerea* and *Pseudomonas syringae* pv. Tomato DC3000	[20]
Overexpression of a novel Chitinase genes *EUCHIT2* enhances resistance to *Erysiphe cichoracearum* DC in tobacco plants	[21]
Overexpression and down-regulation of barley Lipoxygenase *LOX2.2* affects jasmonate-regulated genes and aphid fecundity	[22]
The protease inhibitor *Ci2c* gene induced by Bird Cherry-Oat aphid in barley inhibits Green Peach aphid fecundity in transgenic *Arabidopsis*	[23]
Overexpression of *HvIcy6* in barley enhances resistance against *Tetranychus urticae* and entails partial transcriptomic reprogramming	[24]
Prospects of understanding the molecular biology of disease resistance in rice	[25]
